# The Peri-Saccadic Perception of Objects and Space

**DOI:** 10.1371/journal.pcbi.0040031

**Published:** 2008-02-15

**Authors:** Fred H Hamker, Marc Zirnsak, Dirk Calow, Markus Lappe

**Affiliations:** 1 Institute of Psychology, Westfälische Wilhelms-Universität Münster, Münster, Germany; 2 Otto-Creutzfeldt Center for Cognitive and Behavioral Neuroscience, Westfälische Wilhelms-Universität Münster, Münster, Germany; University College London, United Kingdom

## Abstract

Eye movements affect object localization and object recognition. Around saccade onset, briefly flashed stimuli appear compressed towards the saccade target, receptive fields dynamically change position, and the recognition of objects near the saccade target is improved. These effects have been attributed to different mechanisms. We provide a unifying account of peri-saccadic perception explaining all three phenomena by a quantitative computational approach simulating cortical cell responses on the population level. Contrary to the common view of spatial attention as a spotlight, our model suggests that oculomotor feedback alters the receptive field structure in multiple visual areas at an intermediate level of the cortical hierarchy to dynamically recruit cells for processing a relevant part of the visual field. The compression of visual space occurs at the expense of this locally enhanced processing capacity.

## Author Summary

Early in the vertebrate lineage fast movements of the eye, called saccades, developed. This improvement in spatial direction selectivity has been achieved at a cost to handle a sequence of different views. Recent experiments showed that the brain uses its knowledge about the upcoming eye movement to guide perception prior to the next saccade. They revealed an improved recognition of objects at the saccade target, a change of receptive fields, and a mislocalization of briefly flashed stimuli towards the saccade target. We here offer a novel, unifying explanation for these phenomena and link them to a common neural mechanism. Our model predicts that the brain uses oculomotor feedback to transiently increase the processing capacity around the saccade target by changing the receptive field structure in visual areas and thus, it links the pre-saccadic scene to the post-saccadic one. A briefly flashed stimulus probes this change in the receptive field structure and demonstrates a close interaction of object and spatial perception.

## Introduction

Our visual experience is derived from a multitude of rapid scanning gaze shifts called saccades. However, perception is even more tightly coupled to saccades than by the mere selection of gaze position. This can be observed in at least three dynamic phenomena that occur time-locked to an upcoming saccade. First, in dual-task experiments that require discrimination of an object and the execution of a target-directed saccade, it has been observed that visual discrimination is best when the discrimination stimulus is located at the saccade target [1,2]. Second, receptive fields dynamically change their position and shape. In area V4, receptive fields tend to shrink and shift towards the saccade target [3]. In many other areas such as V3a, LIP and FEF, a receptive field translation along the saccade vector has been described [4–7]. The latter observation is commonly referred to as remapping, since neurons begin to fire towards a stimulus located in the future, post-saccadic receptive field. Remapping has been suggested to play an essential role in visual stability, i.e. our subjective experience of a stable world despite the change of the retinal image with every saccade [4,7]. Third, around saccade onset briefly flashed objects are seen close to the saccade target. This transient distortion of perceptual geometric relationships has been termed peri-saccadic compression [8–11].

There is a general agreement that these phenomena depend on extraretinal signals. Their precise link to particular extraretinal signals, however, is unknown. Among those extraretinal signals is corollary discharge, a copy of a motor command that is sent to the perceptual pathways of the brain. For example, the corollary discharge from the superior colliculus to the frontal eye field encodes the saccade target information, i.e., saccadic eye displacement [12]. Corollary discharge has been primarily associated with the remapping of receptive fields to construct a continuously accurate, retinocentric representation of visual space [4–7]. This remapping of receptive fields, however, would require an extraretinal signal that is distributed across the whole visual space changing the effective connectivity of neurons in retinotopic maps [13]. Another extraretinal signal of the oculomotor system codes for eye position [14]. Information about eye position is crucial for coordinate transformation from a retinocentric to a head-centered reference frame by tuning the response selectivity [15,16]. Localization errors of stimuli flashed in total darkness, known as uniform peri-saccadic shift [17,18], suggest that the eye position signal is erroneous around a saccade [19]. The mislocalization of brief flashes in direction to the saccade target, the peri-saccadic compression [9,11], is virtually not understood, but it has been attributed to a translation in cortical coordinates [20] or a stretching of receptive fields [21]. The facilitated visual discrimination at the saccade target position is usually interpreted as the result of spatially focused visual attention. Presumably, attention-related extraretinal signals during eye movements lead to an enhanced response of neurons that encode a target object selected for saccade [22]. The processing in parietal and frontal cortex has often been associated with attentional spatial selection—the source of spatial attention [23–25].

Here, for the first time, we develop a computational theory of peri-saccadic vision that explains three of the mentioned peri-saccadic phenomena: the enhancement of visual discrimination at the saccade target, the shift of receptive fields, and peri-saccadic compression. Basically, we will demonstrate that these three phenomena can be linked to a single neural mechanism. Our proposed theory assumes that corollary discharge, or more general, a plan to move the eye, is used to transiently boost visual performance at the target location of the saccade immediately before the saccade. While this performance boost is beneficial for visual discrimination peri-saccadic compression is a direct consequence of it, and thus a cost to pay.

## Results

### Model

**Figure 1:**
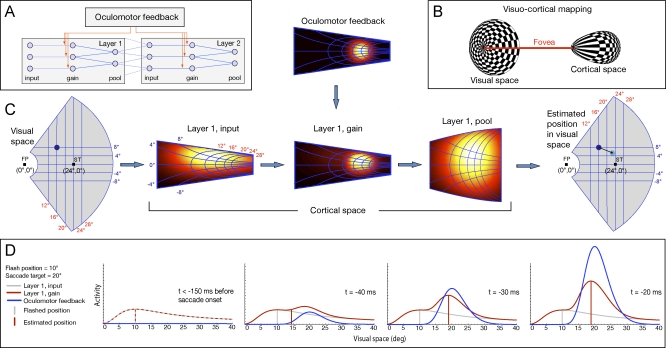
Computational Model for the Oculomotor Modulation of Visual Processing (A) Hierarchical view of visual processing where each cell implements a specific feature detector with a localized receptive field. Each layer consists of three stages (input, gain and pool). The oculomotor system feeds the encoded saccade target position back to multiple layers and increases the gain of the cells prior to spatial pooling. (B) Illustration of the mapping from visual space to cortical space. (C) Detailed view of computations within a single layer. We illustrate the effect on the population response exerted by a peri-saccadically flashed dot at position (16°, 8°) while executing a 24° saccade. The activity distributions in the model are shown in cortical space. The depicted area of cortical space refers to the gray surface highlighted in the visual space. The spatial distortion due to cortical magnification is illustrated by the projection of the grid in the visual space into the cortical space. Using functions of receptive field size, cortical magnification and gaze position, we first determine the cortical population response in the input stage evoked by the flashed dot. The feedback signal determines the gain factor according to its activity profile. The gain modulated population response is distorted towards the saccade target. This population is then spatially pooled to obtain increasing spatial invariance. The perceived position of the stimulus is decoded from the activity in the neural ensemble. (D) Population responses along the horizontal meridian in layer 1, input and layer 1, gain from a flashed dot at position (10°, 0°) before a 20° saccade. Long before saccade onset (*t* < −150 ms) no oculomotor feedback has been built up and the population responses in layer 1, input and layer 1, gain are identical. The decoding of the stimulus position from the population response leads to the true position. At *t* = −40 ms oculomotor feedback is sufficiently strong to distort the population response so that the decoded value is already shifted towards the saccade target. As the occurrence of the flash gets closer to saccade onset, the feedback signal, and thus the gain, increases further and the estimated perceived position is close to the saccade target. However, a further increase of the gain (e.g., flash occurrence at *t* = −20 ms) does not lead to a larger mislocalization.

Early to mid-level visual processing is organized in retinotopic maps in many brain areas. Likewise, saccadic targeting information is organized in visuo-motor maps in cortical (frontal eye field) and subcortical (superior colliculus) structures. In our model, saccade target information is sent back as an oculomotor feedback signal from visuo-motor maps to visual-spatial maps in topographic correspondence and is only available around saccade onset. Each single visual map consists of an input stage with “simple” cells for feature detection, a spatial pooling stage with “complex” cells to obtain increasing spatial invariance [26,27], and an intermediate gain stage at which the oculomotor feedback signal acts. This feedback signal increases the gain of the visual responses of the neurons as observed in electrophysiological studies [25,28]. A hierarchy of visual processing is then obtained by simulating multiple layers where the mechanism of gain modulation acts in each additional layer (Figure 1A).

A visual stimulus initially exerts a corresponding activity hill on the cortical surface of layer 1 (L_1_, Figure 1C, left). The position and shape of this activity hill is determined by the magnification factor (Figure 1B) and the receptive field sizes of the neurons in the simulated area. Prior to an eye movement, activity increases at the location of the saccade target in the oculomotor map (Figure 1C, top). The feedback of this activity distorts the population response of the flashed stimulus towards the saccade target (Figure 1C, center). By assuming that the visual system relies on this population response for stimulus localization, we can decode the perceived position (Figure 1C, right). Figure 1D illustrates the underlying mechanism of mislocalization in detail. Each panel shows the population activities in the input (gray), feedback (blue), and gain (red) layers along a horizontal stretch of visual space. The leftmost panel depicts the case when the flash is presented 150 ms before the saccade. Input and gain layer activities are identical since there is no feedback signal at this point in time. The flash exerts a distribution of activity over the entire population that peaks at the position where the flash was presented (10°). The perceived position is decoded from the distribution of the population activity by a template matching procedure (see “Decoding” in Methods). The decoded position (red vertical line) is identical to the true flash position.

The three panels to the right depict the interaction between feedback and gain layers for flashes presented at three time points before saccade onset (−40 ms, −30 ms, and −20 ms). Over this time course, the feedback signal (blue) rises in strength but is always centered at the saccade target position at 20°. In the gain layer (red curve) the responsivity of the neurons near the saccade target increases and the shape of the population activity is distorted. The decoding of the perceived flash position shifts the perceived position (red vertical line) gradually away from the true position (gray vertical line) and towards the saccade target. As the strength of the feedback signal increases as time gets closer to saccade onset, the strength of mislocalization of a flash presented at that particular time increases as well. For flashes presented spatially beyond the saccade target, the mislocalization would be in the opposite direction, and again towards the saccade target. Mathematical details of the model are described in the Methods section.

### Mislocalization of Briefly Flashed Objects

**Figure 2:**
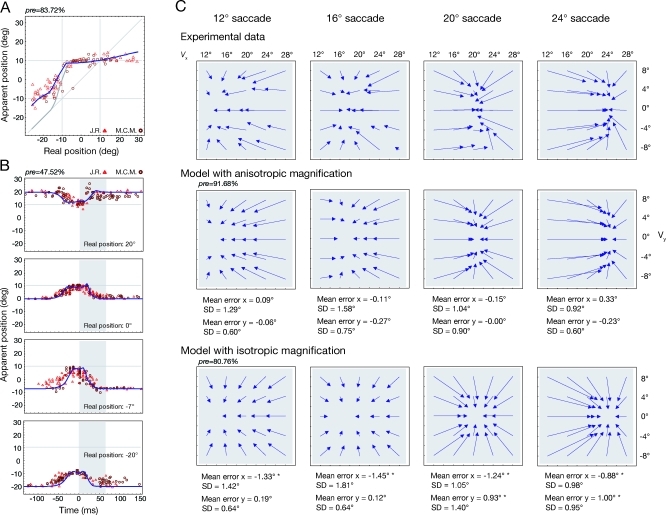
Peri-Saccadic Compression in the Model (A) Spatial range of compression. The data shows the apparent versus real position of flashed bars in the critical phase from −25 to 0 ms before a 20° saccade for two human subjects (data from Morrone et al. [9]). Estimated stimulus location by the model using L_1_ and L_2_ (blue) and only L_1_ (gray). The area around the saccade target is compressed in L_1_ whereas stimuli presented at large distances from the saccade target require another layer (L_2_) with larger receptive field sizes. (B) The time course of compression. The data shows the apparent position of bars presented at four different locations as a function of time relative to saccade onset of two subjects (data from Morrone et al. [9]). The blue line represents the predicted mislocalization of the model. (C) The spatial pattern of compression. The data shows the absolute mislocalization with reference to the true position of a flashed dot randomly chosen from an array of 24 dots for four different saccade amplitudes (data replotted from Kaiser and Lappe [11], who plotted the mislocalization relative to a baseline). Vector origins indicate the veridical flash position and vector endpoints indicate the perceived position around saccade onset. The simulation results show the best fits of models with anisotropic or isotropic magnification. In contrast to the isotropic model, the anisotropic model on average does not significantly deviate from the data (section “Computation of mean errors”). Significant deviations (*p* < 0.05) are indicated by * (two-sided one-sample t-test, *α* = 0.05, *df* = 23).

In the simulations we found that a model with a hierarchy of only two gain modulated layers (L_1_, L_2_) with increasing receptive field sizes is consistent with three particularly relevant experimental data sets of peri-saccadic localization: the spatial range of compression [9], the time course of compression [9] and the spatial pattern of compression [11] (Figure 2). We estimated the goodness of fit by the proportional reduction in error measure (*pre*), which is the reduction in the sum of squared error (SSE) of the data by the model (section “Proportional reduction in error measure” in Methods). The model shows strong compression in the range of ±20° around the saccade target (Figure 2A). The mislocalization originates in L_1_ for stimuli flashed close to the saccade target and in L_2_ for stimuli flashed further away. The effect occurs prior to saccade onset and ceases during the saccade (Figure 2B). Mislocalization of small stimuli occurs also orthogonal to saccade direction (Figure 2C) as the feedback signal acts on the two-dimensional cortical surface. However, only a model of anisotropic cortical magnification in L_1_ results in an adequate fit to the data for all four saccade amplitudes (Figure 2C).

The presented model is the first neural explanation that accounts for the essential data of peri-saccadic compression. Because we put much emphasis on neuroanatomical and physiological details, the model, as defined by the parameter fit to the available data, can provide quantitatively testable predictions. Since most of the relevant anatomical data is not well known in humans we primarily relate to investigations with monkeys.

### The Origin of the Feedback Signal

One prediction is concerned with the origin of the feedback signal. Since the temporal dynamics of compression requires a particular time course of the activity in the oculomotor map, we can use this constraint to predict the origin of the feedback signal. The frontal eye field (FEF) shows a continuum of saccade-related cells ranging from a strong visual to no visual response [29]. Similarly, some cells in the superior colliculus (SC) initially slowly build up their activity and others show a burst of activity only around saccade onset [30]. The movement fields of saccade-related cells in FEF and SC can be closed and open-ended [12,29,30]. Cells with closed movement fields fire only when the saccade amplitude is around the optimum for that cell, whereas with open movement fields a cell continues to discharge also for larger saccade amplitudes. Furthermore, saccade-related cells are clipped, partially clipped or unclipped [30]. The discharge has been classified as clipped if the activity drops close to baseline by the end of the saccade. Although several neurons with open movement fields, primarily of a build-up type, can be found in SC, the majority of burst cells has closed movement fields and a clipped activity profile [30].

We systematically varied the shape and time course and fitted the model to the data, showing the time course and spatial range of compression, by adjusting the other parameters of the model with the constraint that the model remains consistent with the spatial pattern of compression.

**Figure 3:**
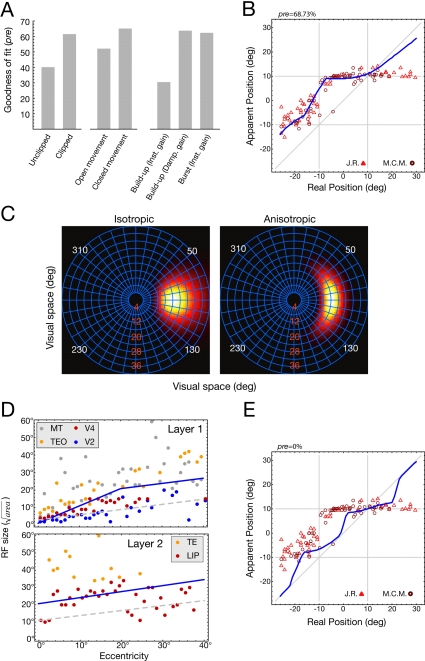
Predicted Source and Shape of Oculomotor Feedback, and Predicted Target Area of Compression (A) Goodness of fit (*pre*) for the time course and spatial range of compression with respect to typical properties of cells in oculomotor areas. Unclipped activity and open movement fields lead to a drop in the goodness of fit. A time course which resembles the firing pattern of burst cells is consistent with the data, whereas build-up like activity with a half maximum value around 46 ms prior to saccade requires a damped gain function in the target area to compensate the early distortion. (B) Effect of open movement fields on the localization of flashed bars in the critical phase from −25 to 0 ms before a 20° saccade. (C) Predicted shape of the feedback signal in visual space for a 20° saccade. The model with anisotropic magnification predicts a shape that is circumscribed for a particular eccentricity but spreads to different angles with constant eccentricity. For comparison, the model with isotropic magnification produces a round shape with a strong spread of the signal to a broader range of eccentricities. (D) Comparison of monkey receptive field sizes with the model prediction (Text S2). The line shows the required minimal receptive field size for each layer. Please note, due to the non-linear spatial pooling in the model, the receptive field values are upper bounds and not mean values. The dots indicate the maximal receptive field size for a particular eccentricity in the respective cortical area as reported in the literature. For the area to be consistent with the model the dots should be close to or exceed the constraint given by the model. Layer 1: The receptive field sizes in V4 are close to the minimal receptive field size of L_1_. Receptive field sizes in MT and TEO are sufficiently large. Layer 2: Both TE and LIP are consistent with the prediction of the model for L_2_. For larger eccentricities, receptive field sizes in LIP are below the lower limit obtained from the model. However, since the critical stimuli in the data (Figure 2A) which constrain the receptive field size in L_2_ were all presented at an eccentricity of less than 20° (in the opposite hemifield than the one where the saccade target appeared) we should not exclude LIP. (E) Effect of small receptive field sizes in L_1_ and L_2_ (dashed lines in [D]) on the localization of flashed bars in the critical phase from −25 to 0 ms before a 20° saccade.

The model predicts that the main contribution originates from cells with closed movement fields and clipped discharge (Figures 3A and S1). Open movement fields systematically reduce mislocalization of stimuli flashed beyond the saccade target, since the feedback signal now shows a weaker spatial gradient for larger eccentricities (Figure 3B). The activity in the oculomotor map should exceed its half-maximum value not earlier than 30 ms prior to saccade onset, which is consistent with the firing pattern of burst cells. However, this value depends on the assumption that the gain is instantaneous, i.e., even a low activity of the cells in the oculomotor map leads to a significant gain increase. We tested the model also with a damped gain function with little increase at the target site for low oculomotor activity (see “Gain Modulation” in Methods), and observed that the half-maximum activity can occur much earlier. Thus, whereas an instantaneous gain function requires that the feedback signal primarily originates in oculomotor burst cells, a damped gain function allows that build-up, and/or visual activity contributes to the feedback signal. In both cases, however, the effective feedback signal would be primarily driven by saccade-related activity, since the early prelude activity would have little impact on the gain.

The feedback signal in the model represents the contribution of many cells which, in experimental data, have to be combined with respect to their firing rate and their movement field to interpolate the activity distribution on the cortical surface [31]. Our prediction about the spatiotemporal properties of the feedback signal could be tested by calculating detailed spatiotemporal activity distributions in the SC and in the FEF for a given a saccade amplitude.

### Cortical Magnification and the Shape of the Feedback Signal in Visual Space

Another prediction is concerned with the shape of the feedback signal in visual space. We modeled the feedback signal as a Gaussian in cortical space similar to collicular neurons with closed movement fields [12,30]. Under this assumption our model predicts an anisotropic magnification in early visual areas. This qualitatively resembles findings in striate cortex of monkey [32,33] and human V1 and V2 [34]. As a consequence of this anisotropic magnification the feedback signal appears elongated in visual space (Figure 3C). This prediction could be tested by estimating the shape of the oculomotor feedback signal in spatially arranged visual maps with fMRI.

### Where Does Visual Compression Occur in the Brain?

Anatomical and physiological investigations revealed widespread connections from the oculomotor system to extrastriate visual areas that list these target areas as candidates for participating in compression. The SC has indirect projections to visual and frontal areas via the thalamus [35]. The FEF is linked with V2, V3, V3a, V4, MT, MST, FST, VIP, LIP, V4t, TEO and TE [36–39]. The FEF projections to these areas appear to be topologically organized in terms of saccadic amplitude [37], as required by the model. A gain increase has been observed in V4 cells after a stimulation of the FEF using currents below the level that evoke a saccade [25]. Subthreshold stimulation in the SC also induces a shift of attention and an increase in visibility at the motor field of the stimulated site [28,40].

In addition to these anatomical and physiological considerations we can formulate stronger constraints on the involved areas by tuning the parameters of the model to the minimal possible receptive field size and compare it to the receptive field sizes of several areas in question (Figure 3D and Text S2). For the strong compression in the spatial range of ±20° around the saccade target (Figure 2A) the model requires at least a receptive field size as observed in areas V4, MT, or TEO, alternatively in V3a as well. The receptive field constraint of L_2_ is consistent with the receptive field sizes found in TE and LIP. Too small receptive field sizes, e.g., at the level of V2 for layer 1 and between 10° and 20° for layer 2, still allow to fit the data from flashed bars close to the saccade target, but effects from those flashed at a larger distance cannot be accounted for (Figures 3E and S2). The reason is that with a small receptive field size the population response becomes too narrow to be affected by the feedback signal so that the spatial range of strong compression is reduced to less than ±10°. Increasing the width of the feedback signal is not a solution. A broader feedback signal would increase the gain of the whole population to a similar degree. However, a mislocalization only occurs when the population is distorted which requires a difference in the gain across the population. Thus, a broader feedback signal would increase the range of compression, but the amount of compression would be reduced (slope of the line through (10°, 10°) in Figure 3E would approach 1).

### Receptive Field Dynamics

**Figure 4:**
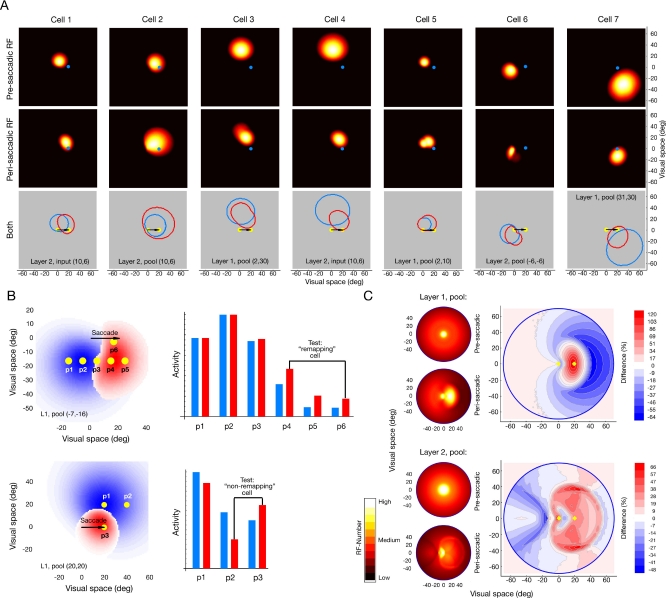
Predicted Receptive Field Dynamics and Capacity Increase (A) Pre- and peri-saccadic receptive fields of seven representative model cells for a rightward saccade of 20° as determined by a half-maximum threshold. Layer of origin and the location of the receptive field centers are given in the lower plots. The yellow and blue dots indicate fixation and saccade target position whereas the arrow shows the saccade vector. (B) Peri-saccadic receptive field changes of two cells. The blue color indicates the pre-saccadic activity profile and the red color the peri-saccadic one. If the peri-saccadic response is larger, it is shown on top of the pre-saccadic one. The yellow dots indicate probe positions and the response to each probe is plotted to the right. The cell in L_1,pool_ with a pre-saccadic receptive field center at (−7°,−16°) remaps with the saccade vector, comparable to electrophysiological observations [7], since the peri-saccadic change in response is maximal around p4. The receptive field does not shift to the saccade target since the response at p6 is lower than the one at p4 and about the same as at p5. The cell in L_1,pool_ with a pre-saccadic receptive field center at (20°,20°) shows by no means remapping. The peri-saccadic response at p3 is higher than the one at p2. (C) The pre- and peri-saccadic processing capacity as estimated by the number of neurons participating in the processing of each part of the visual field. For each position (squared area of 1°) in the visual field we counted the number of selective cells as determined by the mapped receptive field. Due to cortical magnification the pre-saccadic case shows a high capacity in the center. In the peri-saccadic case the model predicts a strong increase around the saccade target. The relative change in processing capacity reveals the areas of increase and decrease in the visual field. In L_1,pool_ the capacity increases around the saccade target and in L_2,pool_ we observe a rough hemispheric effect. The yellow dots indicate the fixation and the saccade target.

We next turn to the predicted receptive field dynamics in the model and their relation to peri-saccadic receptive field changes observed in different brain areas [3,4,6,7]. To determine the receptive fields of model neurons we calculated the spatial borders of the half-maximum response, as is commonly done in neurophysiological experiments. We determined the receptive fields in two conditions, pre-saccadic and peri-saccadic prior to the eye movement. We find combinations of shift, shrinkage and also expansion of receptive fields (Figure 4A). For receptive fields above or below fixation, locations that have been commonly used to investigate remapping, the model shows peri-saccadic shifts of receptive fields similar to those observed in V3a, LIP and FEF [4,6,7,41] (Figure 4B, e.g., a model L_1,pool_ cell with receptive field center at (−7°, −16°)). For cells with receptive fields located above or below the saccade target, the model predictions differ from remapping (Figure 4B, e.g., a model L_1,pool_ cell with receptive field center at (20°, 20°)). Whereas remapping predicts a change into the direction of the saccade (towards p2), for this receptive field, our model predicts a change towards the saccade target (p3), similar to observations made in area V4 [3].

### Transient Capacity Increase Due to Receptive Field Dynamics

Having demonstrated that the proposed model is consistent with the essential data of peri-saccadic compression—the cost side—we now ask for the benefit of oculomotor feedback. A planned saccade increases the gain of cells with receptive fields around the saccade target, similar as observed physiologically [25,42,43]. This increase in gain enhances the sensitivity of the cells and when multiple stimuli are present within a single receptive field, it can bias the competitive interactions among stimuli to suppress the influence of the unattended ones [44,45]. Whereas the link of the processing in oculomotor areas to changes in gain of cells in visual areas is now well established the macroscopic effect of receptive field dynamics is unclear. In order to provide an estimate of the joint effect arising from all receptive field changes, we define visual capacity as the number of cells which process a particular, small part of the visual scene, as determined by their half-maximum response. A higher visual capacity could potentially allow us to reveal finer details of objects and thus facilitate recognition. Due to cortical magnification the capacity of visual processing is not evenly distributed, since most of the cells are devoted to process the central part of the visual scene. To estimate peri-saccadic capacity effects we compared the capacity distribution during fixation with the one around saccade onset (Figure 4C). We observed a capacity increase around the saccade target of more than 100% in L_1,pool_. In L_2,pool_ the model shows a slight increase in capacity almost across the whole visual half-field that contains the saccade target. Thus, our model predicts that areas at an intermediate level of the hierarchy tune their feature detectors to encode aspects of objects located close to the saccade target whereas higher levels are more broadly tuned to the whole visual half-field.

## Discussion

### Mechanisms of Spatial Attention

Our model explains peri-saccadic compression, receptive field shifts, and a visual capacity increase by the same mechanism, i.e., a spatially selective feedback signal that encodes the saccade target. The feedback signal may be provided by the oculomotor system as a corollary discharge [7] or, more abstractly, as a plan to move the eye [45]. This attentional explanation of peri-saccadic compression appears at odds to the explanation by remapping. Although attention can be covertly shifted to locations other than the target location of an upcoming saccade, it is generally accepted that spatial attention is locked onto the saccade target just prior to saccade onset [1,2]. Our model predicts that the effective feedback signal is driven by saccade related activity and thus it supports a premotor view of spatial attention [46]. However, the term premotor has never been clearly defined in the literature. From our point of view, premotor does not imply that the target selection has been finalized. We now know that the FEF and the SC contain a continuum of visuomovement cells from little to strong movement related activity. If these cells are the primary source of feedback there appears sufficient room that the net-signal is movement related in tasks that require an eye movement and that visual cells in the oculomotor pathway contribute to the net-signal and feed back to mid level visual areas, as suggested by a predominantly visual-selection hypothesis of spatial attention [47,48]. Our study supports our previously formulated reentry hypothesis of spatial attention [45,49]. Indeed, the present model is an anatomically more precise implementation of our previous model while dropping some details with respect to the temporal dynamics of competitive recurrent interactions.

It has been suggested that covert attention could be implemented as a planned but not executed saccade [46,50]. If this assumption of covert attention is true, the plan to move the eye would be already sufficient to distort the population response. Thus, our model would predict that it should be possible to observe compression when an eye movement plan is aborted prior to its execution. Moreover, our model would predict a pattern of receptive field changes similar to that shown in the peri-saccadic case. This prediction of our model is supported by the observation that also covert shifts of attention resulted in a shift of V4 and MT response profiles [51,52]. However, a covert attentional signal may be less strong than one immediately before saccade onset and the resulting compression may be small.

### Other Retinal and Extraretinal Factors that Might Affect the Mislocalization of Brief Flashes

Besides compression, caused by oculomotor feedback, other factors might also influence the pattern of mislocalization. In the experiments, subjects have to report the perceived position after the eye movement has taken place. Thus, they must take the saccade into account to avoid a systematic offset in their location estimate and use additional retinal or extraretinal cues, such as an extraretinal eye position [53–57], a prior assumption about stability [58], or the relative distance to stimuli that can be used as landmarks [59–61]. The usage of this additional information allows us to compute an eye-movement invariant stimulus position (with respect to the limits of the additional information) presumably in a second processing stage [62].

If compression relates to an oculomotor feedback signal encoding the saccade target, why is mislocalization in total darkness predominantly characterized by a shift into the direction of the saccade with only little compression [10,18,19]? Whereas under normal conditions the relative distance to landmarks can be used for localization, experiments in total darkness presumably require the usage of an extraretinal eye position signal. While it appears well established that the extraretinal eye position signal does not allow for a perfect on-line correction of the retinal shift, the missing compression appears puzzling. Since there is no obvious reason to postulate the absence of the oculomotor feedback signal in darkness, one would expect compression also in total darkness. However, at least two factors reduce or even diminish compression. First, experiments in total darkness require memory guided saccades or at least saccades with less visual guidance. In memory guided saccades movement related neurons typically fire less vigorously [63] and thus, the gain increase should be reduced. An indirect link between the activity of movement cells and the amount of peri-saccadic compression is also suggested by the correlation of peri-saccadic compression with saccadic peak velocity, since the peak velocity depends on the activity of movement related cells [64]. Second, in our model the gain increase depends on the stimulus strength, consistent with the observation that the magnitude of compression decreases with increasing contrast [65]. Thus, the model predicts weak or diminished compression in total darkness, if stimulus luminance is high. Indeed, cell recordings in V4 and MT revealed that the gain enhancement due to shifts of spatial attention is limited for high contrast stimuli [66,67], although the exact gain function is debatable [68]. It is not clear if our predicted oculomotor feedback signal is identical to the signal causing shifts of spatial attention in those experiments, but the mechanism of gain enhancement might be independent of the source of the modulatory signal. However, for low luminance stimuli the model predicts compression, even in total darkness. We recently tested this prediction experimentally and found compression in total darkness for stimuli with near-threshold luminance [69]. Thus, compression can also be observed in total darkness, as predicted by the model, if stimuli are presented at low visibility.

The process of determining the position in world-centered coordinates may also influence the mislocalization effects observed after saccadic adaptation. After saccadic adaptation, it has been observed that peri-saccadic compression is directed to the adapted end point of the saccade [70], whereas the activity in the SC appears to encode the initial, unadapted location of the saccade target [71] (see however [72,73]). There are two possibilities to reconcile this observation with our model. First, it may be possible that the pre-saccadic compression is directed to the unadapted goal location and that it is subsequently shifted towards the post-saccadic gaze direction by adaptation specific spatial transformations. This is supported by observations of general shifts of perceived visual location induced by saccadic adaptation outside the time interval for compression [74]. Second, more monotonic adaptation techniques could lead to cognitive changes in the saccade plan so that the feedback signal is indeed pre-saccadically directed towards the adapted end point of the saccade. This has recently been shown by observations of mandatory pre-saccadic allocation of attention towards the adapted end point after saccadic adaptation [75].

### The Potential Target Areas of Compression

The model makes clear and strong predictions about the putative involved areas with respect to the receptive field size. We can restrict the origin of the strong compression of ±20° around the saccade target to intermediate levels of the cortical hierarchy. The observed dissociation that much less compression is found for pointing movements with closed eyes than for verbal reports of the perceived position [76,77] can be explained by different pathways for perception and for pointing. Online reaching and pointing movements recruit the “dorso-dorsal stream” [78] consisting of the forward projection V1 to PO to MIP and V2/V3 to V6/V6a to MIP and further to supplementary motor areas [79,80]. This stream has not been reported to receive significant feedback from the lateral FEF [37]. Thus, consistent with observations in MT/MST [81], our model predicts that the encoding of a stimulus position is already distorted in a retinocentric reference system presumably at the levels of V3a, V4, TEO, MT/MST and LIP.

### Receptive Field Dynamics

Fitting the data of peri-saccadic compression predicts a specific pattern of receptive field dynamics. This linkage of psychophysical data to their underlying neural brain processes is a particular strength of our approach. The cells shown in Figure 4A and 4B exemplify the fact that the receptive field effects in our model are dependent on the relative locations of fixation, saccade target, and center of the receptive field. The similarity of our model observations with studies in different brain areas raises the fundamental question about the nature of peri-saccadic receptive field changes. We demonstrated that our model predicts the remapping of receptive fields for receptive field positions that have been commonly used to investigate remapping [4,6,7,41]. For other locations, however, the model is consistent with observations made in V4 where receptive fields tend to shift towards the saccade target and not along the saccade vector [3]. Do V4 receptive field dynamics differ from other areas? For example, does remapping occur primarily in oculomotor-related areas whereas our model describes properties of areas involved in the computation of object identity? Or is remapping not homogeneous across visual space, but a special case that applies only within a certain part of the visual field? A non-homogeneous remapping could reconcile the different observation made in V4. No study has yet systematically addressed this question. Such systematic investigations of 2D receptive field dynamics in different brain areas are required, specifically in those which receive oculomotor feedback, e.g., area V4, MT, MST, V3a, TEO, LIP and VIP.

### The Change in Processing Capacity and its Relation to Object Recognition

Our model suggests that the transient receptive field changes serve an increase in processing capacity around the saccade target. This phenomenon has not been an integral part of earlier attention theories and offers an alternative to the common attentional spotlight metaphor. According to the spotlight metaphor the width of the focus must be properly tuned to the size of the object to which attention is directed, since processing outside of the spotlight is weak. A change in the processing capacity may offer a more robust solution since more neurons are available to process details of the object at the saccade target. Under certain assumptions one can show that the increase of the number of cells within a population improves the accuracy of coding [82,83]. However, an improvement in object recognition must be investigated with more elaborated future models. Indeed, if we took into account that each cell in the model layers is sensitive for a specific feature at a certain position in the visual field, the model would predict a shift in the spatial arrangement of feature detectors at an intermediate level of recognition. This suggests that the structure of objects, as determined by the feature detectors in each brain area, remains uncompressed but the position of an object is subject to change. This is consistent with the observation that the shape of a single object is not or much less distorted [84,85].

### The Subjective Experience of a Stable World

What mechanism is then responsible for the perception of a stable word? Although remapping has been suggested to lead to the perception of a stable environment [4,7], a global anticipatory shift of receptive fields might not be necessary. Perhaps the brain does not even attempt to maintain a continuous retinocentric representation of visual space [86,87]. In this regard, compression is not used for a correction of peri-saccadic artifacts to realize a stable, spatially correct representation of the external world. We rather suggest that the anticipatory processing of the object of interest at the saccade target position leads to the perception of a stable world, since we already deal with the object of interest before we even look at it [88]. Several findings, such as saccadic suppression [21] or saccadic suppression of image displacement [89] suggest that, under normal viewing conditions, we make little use of the retinal image during eye movements, but we primarily use information in the pre- and post-saccadic scenes [59,61]. Thus, oculomotor feedback could be essential to link the pre-saccadic representation with the post-saccadic one. First, oculomotor feedback reactivates the pre-saccadic representation of a stable stimulus at the saccade goal which otherwise would decay close to baseline [43,90]. Second, a strong increase in the visual capacity around the saccade target may reveal details of the object that will otherwise only be seen when the eyes land. While the representation of space and thus the perception of a stable world remains an open issue, for now a useful working hypothesis is that the brain deals with the pre-saccadic representation while the eyes move. The peri-saccadic change in the firing pattern of many early to mid-level cells could be negligible, since the pre-saccadic representation is cognitively linked with the post-saccadic one by attention and processing is focused on specific aspects of the scene.

### Conclusion

In conclusion, each saccade is accompanied by an oculomotor feedback signal, which is conveyed to mid-level visual areas and enhances the gain of cells located around the saccade target. Such gain increases lead to an advantage for the processing of stimuli located at or near the saccade target such that they are represented more actively. Moreover, the population response for stimuli presented around the saccade target is distorted. From the viewpoint of a single cell, oculomotor feedback increases its gain and alters its receptive field. On a macroscopic level the changes in receptive field size and location dynamically increase the processing capacity around the saccade target. The spatial mislocalization occurs whenever the brain must rely on the distorted population response in this period to generate a holistic impression of an object in space.

## Methods

### Mathematical description of the model.

Our model of peri-saccadic perception aims at linking psychophysical data with their underlying brain processes. Although the general idea of the model is very simple, our emphasis on certain neuroanatomical and physiological details requires some advanced techniques. The consideration of important neuroanatomical and physiological details has several advantages over more simple approaches (compare [20,90]). This added detail does not primarily serve a better fit; it rather provides a more meaningful constraint. Moreover, the model has more predictive power in the sense, that the obtained parameters are meaningful with respect to a specific cortical function.

The model consists of two visual, hierarchically organized layers L_1_ and L_2_. The computation in each layer is divided into three stages. The first stage represents the input from earlier areas. The second stage implements a gain modulation of the input, and the third stage pools the responses to obtain increasing spatial invariance.

The spatial pattern of compression obtained from a flashed small dot randomly chosen from an array of 24 dots [11] cannot be reproduced by a model in which the feedback signal is defined as a Gaussian in visual space. The consideration of cortical magnification not only allows us to quantitatively fit the data, it also provides us an estimate of the shape of the feedback signal in visual space. Moreover, it affects the direction of dynamic receptive field changes. Magnification changes along with spatial pooling to account for the fact that higher areas typically show a less pronounced magnification at the fovea. The cortical space is mathematically described as a curved surface [91]. The shape of this surface depends on the changes in cortical magnification along the horizontal meridian of the visual field *M_p_*(*ɛ*) and along isoeccentric rings *M_e_*(*ɛ*), where *ɛ* denotes eccentricity (see section “Cortical space” for details). Let *V* be the visual space, 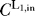 the cortical space of the input into L_1_, 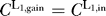 the cortical space of the gain modulated stage and 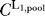 the cortical space of L_1,pool_. We use Gaussian functions to model the receptive fields. Let 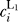 ∈ *V* be the position of the receptive field center, i.e. the point in visual space which maximally activates the cell *i* in L_1_. 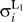 determines the width of the receptive field as a linear function of eccentricity (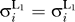 (*ɛ*)). Let *p*_s_∈*V* be the position of the flashed stimulus in the visual field. For simplicity, we ignore the stimulus width. The activity of a given L_1,in_ cell is then defined by

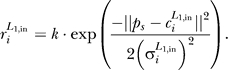



Note that 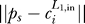 denotes the distance between the receptive field center and the stimulus position. *k* is a constant which relates to the contrast/luminance of the stimulus at the time of the flash. After saccade onset the retinal position of the stimulus is computed according to the position of the eye in space. Our model of eye movements is given in section “Simulation of eye movements.”

Formally, the gain modulated response 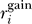 can be described by a sensitivity increase of a cell *i* to its input 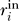 dependent on the oculomotor feedback signal 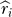 . As derived in section “Gain modulation,” the activity of a given L_1,gain_ cell *i* is defined as a function of the input 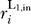 , the gain and a term which normalizes the activity:

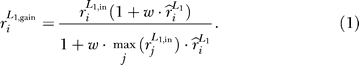



The weight factor *w* is equal for all layers. 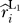 denotes the feedback signal. The feedback signal could have its origin in an oculomotor map in which an activity hill is built up around the target location of a planned eye movement. The oculomotor map is fully connected with L_1_ and L_2_. The feedback signal from the oculomotor map to L_1_ and L_2_ is determined as a Gaussian in cortical space which changes in amplitude through time:

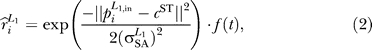
where 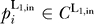 denotes the cortical position of the cell *i* in L_1,in_ and *c* ^ST^ ∈ 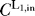 denotes the center of the feedback signal in cortical coordinates. The center of the feedback signal could be an independent variable linked to the saccade plan, but in all simulations performed, we assume that it is equal to the experimentally defined saccade target. 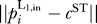 denotes the distance between the position of a given L_1,in_ cell and the saccade target in cortical space. Our model of cortical space is given in section “Cortical space” and the computation of distance in cortical space is explained in section “Distance measurement on the cortical surface.” 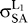 is the saccade amplitude dependent (SA ∈(12°, 16°, 20°, 24°}) width of the feedback signal. The assumption of a gradual spatial decrease of the feedback signal relative to the saccade target is supported by a recent observation in V4 using below threshold microstimulation in the frontal eye field [44]. In this study, the increase of the separation between the saccade endpoint (as determined by above threshold stimulation) and the stimulus in the receptive field of a cell resulted in a decrease of the enhancement effect. Thus, the closer the stimulus is to the saccade target, the stronger is the gain increase by microstimulation. Please note that the predictions of the model do not depend on a retinotopic projection as long as the connections between oculomotor areas and the visual areas correspond with each other in visual space.

As far as the temporal characteristics *f*(*t*) are concerned, the activity hill should increase for *t* ≤ 0, and decrease for *t* > 0, where 0 represents the onset of the saccade. Thus, the strength of the feedback signal is maximal for a stimulus flashed at saccade onset. The center of the feedback signal moves with the eye, i.e., it remains at its original position in a retinocentric coordinate system. In order to test how far the feedback signal relates to the typical time course of movement-related cells in the frontal eye field or the superior colliculus, we systematically varied the time course and shape of the signal (Text S1). For example, for *f*(*t*) we used an exponential:

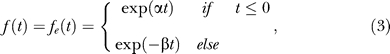
and Gaussian function:

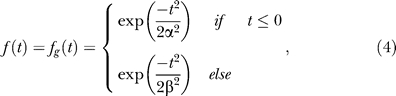
where *α* determines the increase and *β* the decrease of the activity over time in both cases.

We do not explicitly simulate the visual latency of a stimulus to reach an area of interest nor the delay of the oculomotor signal. If we assume that the latency of a stimulus to reach V4 is longer than the delay of the oculomotor signal to reach V4, our feedback signal should have its peak after saccade onset. However, a flashed stimulus has a neural persistence of 100–150 ms, which implies that it is not necessary that the feedback signal must be present at the very first response. Thus, the time course of our feedback signal will appear still plausible if we consider visual latency and persistence.

The activity 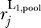 of a given L_1,pool_ cell *j* is determined by pooling the gain modulated input activities of the respective L_1_ cells. The classical receptive fields of L_1,pool_ cells are incorporated into the model using Gaussian functions. The activities of L_1,pool_ cells are weighted with respect to the distance 
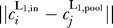
 in the visual space between the receptive field center of the cell *i* in L_1_ input 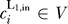 and the receptive field center of the L_1,pool_ cell 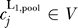 . These weighted L_1_ cell activities are then spatially pooled using a max operation [92]

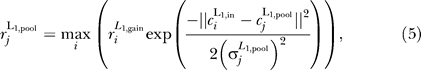
where 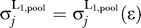 relates to the width of the receptive field. These receptive field kernels only indirectly define the final receptive field size of each layer. Thus, our given estimates of the receptive field size were obtained from mapping the receptive fields (section “Mapping of receptive fields”). The activities in L_2_ are computed equivalently using Equations 1–5.

With the above methods we can calculate the population response of model neurons to a visual stimulus. In order to relate the perceived stimulus position of the model to experimental data, we decoded the population response with respect to location (section “Decoding”). Our model predicts that a compression of space perception is caused by a local increase in the processing capacity. This might appear as a paradox to some readers, since if the receptive fields shift towards the saccade target, the same position in space now activates neurons with receptive fields farther from the saccade target. However, this does not lead to an expansion of space, since the change of the receptive fields is not uniform and other neurons with receptive fields closer to the saccade target still respond to the stimulus. In addition, the neurons closer to the saccade target increase their sensitivity more than the ones farther away. Thus, across the whole population the neurons closer to the saccade target vote stronger, even if the ones farther away shift their receptive field closer to the saccade target.

In order to focus on the localization error predicted by the oculomotor feedback, we do not consider any additional errors due to the mapping of a retinal coordinate system into a world centered coordinate system [19,56]. Thus, we add the position of the eye *θ* (section “Simulation of eye movements”) to the estimated stimulus position in retinal coordinates 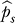 to obtain the estimated stimulus position 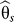 in the world-centered space 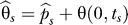 .

We simulated more than 48,000 cells per layer which were equally distributed in cortical space up to 70° eccentricity.

We iteratively determined the parameters and the number of layers in the model (section “Fitting procedure and parameters of the model”) from three particularly relevant experimental data sets: the spatial range of compression [9], the time course of compression [9] and the spatial pattern of compression [11].

### Model details: Gain modulation.

In our model we use static neurons. We here derive the equation of gain modulation for static neurons from an equation used for dynamic neurons. Let us therefore assume, we have a set of gain-modulated neurons. The firing rate of each neuron can be described by a differential equation [45,92]

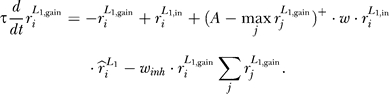



Such a gain function is motivated by several electrophysiological studies which have shown that feedback signals have a modulatory influence [25,93] and it has successfully been applied to model the effect of feedback connections on feedforward processing [45]. The term 
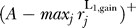
 ensures that the efficiency of the feedback signal depends on the activity of the postsynaptic cell population. If the maximal firing rate exceeds the value *A*, the feedback signal 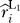 no longer affects the gain. This term has been shown to be consistent with a multiplicative contrast gain modulation as observed in several single cell recordings [94].

When we numerically compute the firing rate and set the weight of the dynamic inhibition among the cells to *w_inh_* = 0, the change of activity 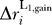 in each time step is

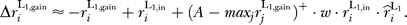



**Figure 5:**
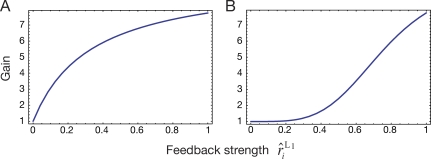
Gain of a Neuron *i* with Respect to the Feedback Strength 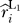 for an Input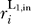 = 0.1 The gain is equal to 1, if no feedback signal is present. An increase of the feedback signal enhances the gain of a neuron. (A) Instantaneous gain function. (B) Damped gain function.

When we ensure that 
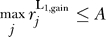
 and further approximate

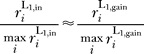
we obtain for the equilibrium a non-recursive equation for the firing rate of the gain modulated neurons (Figure 5A):

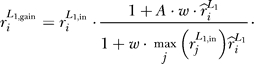



This equation for the firing rate of a static, gain-modulated neuron is of course not equal to the dynamic, recursive solution, but it captures the essentials as verified by simulations.

We alternatively used the following damped gain function to explore the source of the feedback signal (Figure 5B):

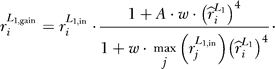
The damped gain function only leads to small changes in gain for low feedback activity.

### Model details: Cortical space.

Neurophysiological findings in monkeys and humans indicate that central parts of the visual field are processed by a greater amount of cortical tissue as compared to peripheral parts [95,96]. The amount of cortical tissue, which processes one degree of the visual field, is termed the cortical magnification factor and is usually denoted in millimeter per degree [97].

For the mapping of the visual field *V* into cortical space *C* a procedure by Rovamo and Virsu [91] has been used according to which the cortical space is a topologically isomorphic distortion of the visual space, i.e., a transformation of a sphere. The visual space is described in spherical coordinates (*ɛ*, *φ*) and the cortical space is described in cylindrical coordinates (*r*, *z*, *θ*). Note that the angle *ɛ* is the eccentricity generating the meridians and the angle *φ* generates the circles of constant eccentricity. To obtain the cortical representation *C* of the visual field *V*, the sphere is transformed according to the two cortical magnification functions *M_p_*(*ɛ*) and *M_e_*(*ɛ*). *M_p_*(*ɛ*) describes the changes in cortical magnification along the meridians of the visual field and *M_e_*(*ɛ*) along the circles with constant eccentricity. If both functions are equal, the cortical magnification is isotropic, i.e. at each location in the visual field magnification along a circle of constant eccentricity is equal to magnification along a meridian. The magnification function *M_p_*(*ɛ*, *φ*) along the meridian is defined by

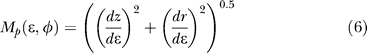
and the magnification function *M_e_*(*ɛ*, *φ*) along the circles of constant eccentricity is defined by

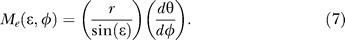



It is assumed that the cortical space is rotationally symmetric (*θ* = *φ*), i.e., the magnification functions do not depend on *φ*. Solving Equations 6 and 7 yields the complete transformation rule

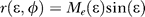
and

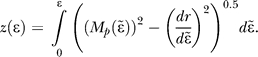



**Figure 6:**
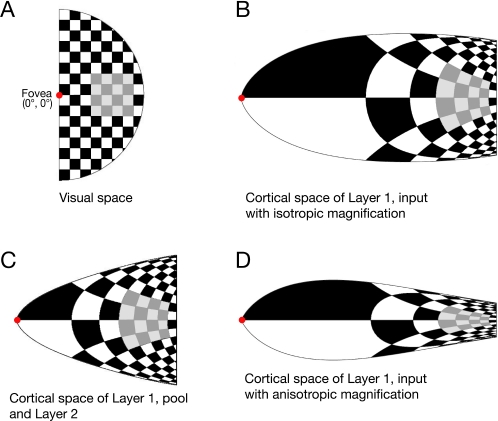
Visual Hemifield and the Respective Side View of the Different Cortical Model Surface from the Fovea up to 32° Eccentricity The center of the visual field, i.e., the fovea, is indicated by the red dot. Each checkerboard element is 4° by 4° in visual space. The gray shaded part indicates the area where the dots in the experiment of Kaiser and Lappe [11] were presented. (A) Visual space. (B) Layer 1, input with isotropic magnification (*M_p_* = *M_e_*). (C) Layer 1, pool and Layer 2. (D) Layer 1, input with anisotropic magnification (*M_p_* > *M_e_*).

Figure 6 shows the cortical space of L_1_ and L_2_. Please note that the difference in size (surface) between the isotropic and anisotropic case is not relevant for the different predictions. The main factor leading to the stronger asymmetry of the compression pattern are the longer distances along the rays compared to the ones along the circles. The degree of overrepresentation around the fovea is not crucial for the results obtained. Please note that the assumed anisotropy across the whole visual field is a simplification. We do not claim that the whole human visual field is subject to anisotropy.

### Model details: Distance measurement on the cortical surface.

Since we describe the oculomotor feedback signal as a Gaussian in cortical space, the distance between the center of the signal and the cortical position of each cell is required. In order to compute the distance one has to consider that the cortical space is a curved surface and the distance between two points is the length of the geodetic line connecting the two points. The geodetic line is the solution of the following variation problem. Let *s* be a real number running from 0 to 1, *g*(*x*_1_, *x*_2_) be the metric tensor of the surface with respect to the local coordinates (*x*_1_, *x*_2_), and let (*x*_1_(*s*), *x*_2_(*s*)) be a path connecting the points (*x*_1_(0), *x*_2_(0)) and (*x*_1_(1), *x*_2_(1)). Finding the geodetic line is done by minimizing

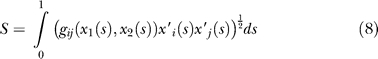
by variation over the possible paths (*x*_1_(*s*), *x*_2_(*s*)) connecting the points (*x*_1_(0), *x*_2_(0)) and (*x*_1_(1), *x*_2_(1)). *S* is the length of the path, 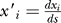 and 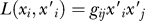 is called the Lagrange function of the variation problem. The solution of this variation problem is equivalent to the solution of the system of differential equations:

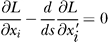
with respect to the boundary conditions (*x*_1_(0), *x*_2_(0)) and (*x*_1_(1), *x*_2_(1)). The local coordinates describing the cortical space are the eccentricity *x*_1_ = *ɛ* generating the meridians and the angle *x*_2_ = *φ* yielding the circles of constant eccentricity. The metric tensor *g*(*ɛ*, *φ*) can directly be calculated from Equations 6 and 7 as shown in the following.

The infinitesimal path-length on the cortical space is in cylindrical coordinates:

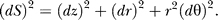



Using Equations 6 and 7 we obtain:

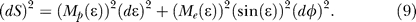



Recall Equation 8, the components of the metric tensor *g*(*ɛ*, *φ*) can be directly taken from Equation 9:

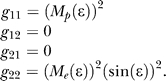



In terms of the metric tensor *g*(*ɛ*, *φ*) one obtains the Lagrange function:

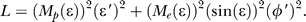



This yields a system of differential equations of second order:

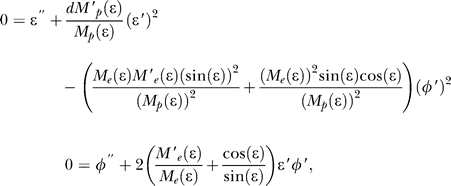
where 
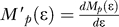
 and 
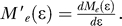


Since this system of differential equations has no analytical solution, it was solved numerically with respect to the boundary condition, i.e., the two points in visual coordinates (*ɛ*(0) = *ɛ*_0_, *φ*(0) = *φ*_0_, *ɛ*(1) = *ɛ*_1_, *φ*(1) = *φ*_1_). When two points are in different hemispheres the shortest path through the fovea has been used. Thus, for each simulated cell we computed its distance to the saccade target on the cortical surface.

### Model details: Simulation of eye movements.

Since the subjects' eye position is not always available, we took a more general approach and simulated the time course of each saccade by approximating its velocity profile using a sixth-order polynomial

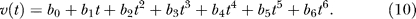



Given the following constraints:

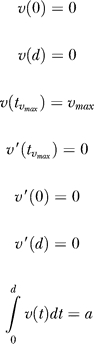
it is possible to find a unique solution for the seven free parameters. If the amplitude *a* of a saccade is given, we have to determine the duration *d* of a saccade, the maximal velocity *v_max_* and the point in time 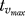 where the velocity reaches its maximum. The duration of each saccade was obtained by *d* = *d*_0_ + *d*_1_ *a* [98]. Consistent with Becker [98], who reported a range of 20–30 ms for *d*_0_ and a range of 2–3 ms per degree for *d*_1_, we set *d*_0_ = 25 ms and *d*_1_ = 2.5 ms per degree. Knowing the duration of a saccade, the mean velocity 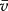 is given by

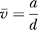
and the peak velocity of a saccade is

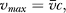
where *c* denotes the ratio of the peak velocity to the mean velocity, i.e., 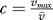 . Becker [98] approximated *c* with a constant value of *c* = 1.65.

Finally, we have to determine 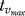 . Takagi et al. [99] defined the skewness *S* of the velocity profile by the ratio of the acceleration phase to the duration of the saccade. For rightward saccades they estimated the following linear regression equation

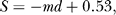
where 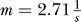 . With *S* it is possible to determine

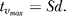



**Figure 7:**
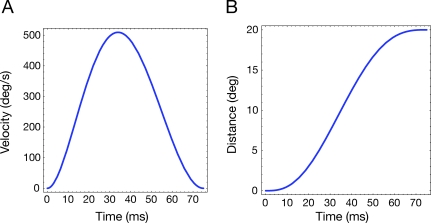
Simulated Saccade (A) Velocity profile a of simulated 20° saccade. (B) Position of the eye relative to the FP at 0°.

After determining the parameters of Equation 10 with respect to the constraints for each saccade amplitude (Table 1), we obtain the velocity and the path of the eye movement (Figure 7). The angle *θ* the eye moves within a time interval [*t*_1_, *t*_2_] is given by the integral

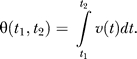

*θ* is then used in the model to update the retinal eccentricity of a flashed stimulus during a saccade. Assuming a stimulus is flashed at time *t_s_* at position *θ_s_* (*t* = 0 denotes saccade onset), the eccentricity *ɛ_s_* of the stimulus with respect to the actual eye position is then

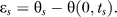



**Table 1:**
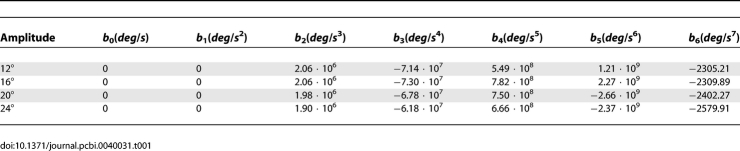
Overview of the Parameters for the Simulation of the Eye Movement

### Fitting procedure and parameters of the model.

Our neurocomputational model of peri-saccadic perception has been parameterized using mathematical functions to describe the anatomy and the neural dynamics, such as the shape and timing of the feedback signal and the receptive field size over eccentricity. Nevertheless, we have unknown parameters which could not be determined by other independent investigations (Table 2). We estimated these unknown parameters to fit the model with the data.

**Table 2:**
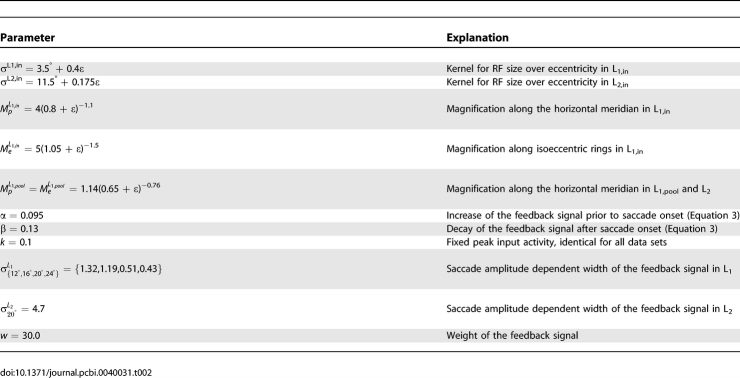
List of All Model Parameters

We simulated the exact time course of the perceived stimulus position given the time and position of the flashed stimulus. In order to relate the model to data taken from a particular time window, we calculated a mean time value 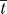 from all data points in the time window. From the data showing the spatial pattern of compression [11] we obtained *t* = 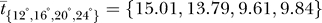 ms as being used for *f*(*t*) in Equations 3 or 4. We considered all data points from 0–25 ms for the 12° and 16° saccade amplitude and from 0–20 ms for the 20° and 24° saccade amplitude. The size of the window was chosen to obtain a sufficient number of trials in the time bin where the effect of compression is strongest. From the data showing the spatial range of compression in the critical phase from −25 to 0 ms [9], we obtained a mean time value of 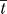 = − 11.38 ms.

With respect to receptive field size, only the receptive field sizes in the input of each layer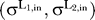 are constrained by the data, since the neural population in this layer provides the input for the gain modulation and thus the degree of distortion. The data provides only little constraints about the overall magnitude of magnification, as verified by simulations with different magnification factors. However, since the ratio of cortical magnification along rays (*M_p_*) to magnification along circles (*M_e_*) has turned out to be relevant for fitting the spatial pattern of compression (*M_p_* > *M_e_*), we have to determine specific values. To reflect the input of earlier stages the cortical magnification along the rays in L_1,in_ 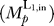 was chosen similar to the magnification in area V2 of monkey and the magnification in L_1,pool_ 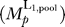 similar to monkey MT and V4. Since we do not know direct measurements of cortical magnification in higher areas we set 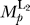 identical to 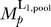 . The magnification along the rings of constant eccentricity 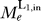 could either be identical to 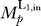 (isotropic condition) or different (anisotropic condition). In all other model parts, magnification is isotropic. In the anisotropic condition, we roughly determined 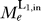 to obtain a sufficient fit of the data showing the spatial pattern of compression.

After running these preliminary simulations to obtain plausible initial values, the fitting procedure was performed in two steps. In the first step, we started with small receptive field sizes as well as with a small value for the strength of the feedback signal *w* and iteratively increased them by allowing adjustments to the initial values of the other parameters 
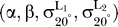
 on the data from Morrone et al. [9]. Besides determining the RF sizes, this fitting process resulted in the final values of *α*, *β*, the strength *w* of the feedback signal and the width of the feedback signal in L_2_ 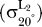 for a 20° saccade amplitude. In the second step, we obtained the final values of the saccade amplitude dependent feedback width (
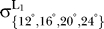
 ) on the data from Kaiser and Lappe [11]. This was done by minimizing the sum of the absolute errors between model and data, i.e., the absolute differences in the *x*- and *y*-direction for each saccade amplitude, as a robust estimation procedure [100]. The obtained value for the width of the feedback signal 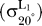 was then also used in the simulation of the data from Morrone et al. [9]. Thus, all data was fitted with a single parameter set.

### Computation of mean errors.

Mean errors between data and model for the spatial range of compression (Figure 2C) were computed as follows: for each of the eight conditions, i.e., each model specification (isotropy versus anisotropy) and each saccade amplitude (12°, 16°, 20°, 24°), the differences between the vector endpoints of the perceived and the predicted flash positions were obtained for the *x*- and *y*-directions, yielding to a total of 48 differences (24 differences in the *x*-direction and 24 differences in the *y*-direction) for each condition. Then, the mean error for both the *x*- and *y*-directions was obtained by computing the respective arithmetic mean. Negative values indicate an undershoot of the model, i.e., the theoretically predicted component is smaller than the empirically obtained compression.

We tested separately for the *x*- and *y*-directions for each saccade amplitude if on average each model deviates statistically significant from the data (two-sided one-sample t-test, α = 0.05, *df* = 23). For the isotropic model all mean errors reach statistical significance (*p* < 0.05) except the error in the *y*-direction for the 12° and 16° saccade. For the anisotropic model none of the mean errors reaches statistical significance (*p* > 0.05).

### Proportional reduction in error measure.

In order to quantify the model fit we used the following proportional reduction in error measure

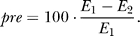



*E*_1_ is simply the sum of squared error (SSE) of the data with respect to a particular empirical mean value and *E*_2_ is the SSE with respect to the corresponding model predictions. If *E*_2_ ≥ *E*_1,_ *pre* was set to zero. Since we have *i* = 1...13 pairs of (*E*_1_;*E*_2_) (8 of the spatial compression pattern, 4 of the time course, 1 of the spatial range), aggregated *pre*-measures where obtained by summing up the respective *E*_1i_ and *E*_2i_ so that

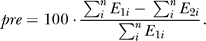



To exclude the apparent shift in baseline from the measurement, we additionally determined pre*-measures of bars flashed at 20° and −20° for which errors (*E*_1_ and *E*_2_) were computed using data points only in the period before *t* = 40 ms.

### Mapping of receptive fields.

For a comparison of the model receptive fields with mapped receptive fields in cortical cells we have to apply the same methods. To approximate the receptive field size, one-dimensional activity profiles were obtained by presenting a point stimulus in steps of 1° along the horizontal meridian. Since the size of a given receptive field kernel only depends on the eccentricity of the receptive field center in visual space, only cells of one hemisphere with centers along the horizontal meridian were included into the mapping to speed up the procedure. As commonly done in electrophysiology the obtained activity profiles were fitted using a Gaussian, yielding a set of receptive field widths defined by the *σ* of the respective Gaussian. According to Albright and Desimone [101] who approximated the size of receptive fields by Gaussian functions, the ratio of the width (*σ*) to the manually mapped width of the receptive fields is about 0.5. Thus, in order to convert the set of the estimated L_2,in_ receptive field width into the usually used 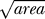 , each entry of the set is multiplied by a factor of 2. This converted set was then fitted with a linear function to obtain the final description of the receptive field size in L_2,in_.

### Receptive field dynamics.

We determined the receptive field dynamics of the L_1,pool_, L_2,in_ and L_2,pool_ cells with a half-maximum response threshold in two conditions, pre-saccadic and peri-saccadic (*t* = 0). The half-maximum response threshold is a common method to analyze physiological data [3]. The pre-saccadic receptive field was mapped without any feedback and the peri-saccadic receptive field with maximal feedback strength using dot stimuli presented in the visual field in steps of 4°. The obtained activity profile was then normalized to the maximal activity and interpolated to a resolution of 1°. The receptive field of a given cell is then defined as the area in visual space in which the activity exceeds half of the maximal activity of this cell.

### Decoding.

The model provides us a population response with respect to a flashed stimulus. In order to compare the output of the model with the data we have to determine the perceived stimulus position by decoding the population response with regard to spatial position. Since the model is deterministic and noiseless, decoding approaches based on probability distributions such as Bayesian inference are not appropriate. Thus, we directly use the firing rates *r_i_* and assume that a number of active cells *N* participate in encoding the stimulus location *p_s_*. **r** = (*r*_1_, …, *r_N_*} can be considered as a vector in the *N*-dimensional space of neural responses. The unmodulated (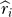 = 0) activity distribution resulting from the presentation of a stimulus at the location *p_s_* is used as a template **f** = **r**(*p_s_*) to which the distorted 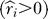 population **r** is compared. The estimated position 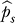 (in retinocentric coordinates) is the one for which the angle between the two vectors **r** and **f** is minimized [102], which is equivalent to

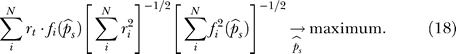



This measure is particularly useful, since it tolerates the absolute increase in firing rate through the gain modulation. Please note our results are not qualitatively dependent on this particular method of decoding.

## Supporting Information

Figure S1 Variation of the Oculomotor Feedback Signal in Time and Shape (559 KB PDF)

Figure S2 Variation of the Receptive Field Sizes (185 KB PDF)

Text S1 The Origin of Oculomotor Feedback in the Brain (74 KB PDF)

Text S2 Estimates of the Cortical Areas Involved Based on Receptive Field Size (62 KB PDF)
