# Supplementary material for: The Peri-Saccadic Perception of Objects and Space
Source: PLoS Comput Biol. 2008 Feb 15;4(2):e31. doi: 10.1371/journal.pcbi.0040031 (PMC2242822; doi:10.1371/journal.pcbi.0040031)
Supplement: Supplementary file 1 [file pcbi.0040031.sd001.pdf]

Supporting material for

Fred H. Hamker, Marc Zirnsak, Dirk Calow & Markus Lappe: The peri-saccadic perception of objects and space

Text S1: The origin of oculomotor feedback in the brain

Since the predicted mislocalization of stimuli flashed at different times relative to saccade onset critically depends on the time course $f(t)$ of the cells in the oculomotor map, we varied the time course and shape of the oculomotor feedback signal to test if the model is constrained by the typical behavior of cells in oculomotor areas such as SC and FEF.

From Figure S1A to S1D we increased the timing from a late, sharply bursting response to earlier burst or even build-up activity. We observed that the data can be fitted by either simulating the time course using an exponential (Figure S1A) with a half maximum value of 7.4 ms before and 5.3 ms after saccade (Equation 3, main text), or a Gaussian function (Figure S1B) with a half maximum value of 21.2 ms before and 14.1 ms after saccade (Equation 4, main text).

A shift to earlier build-up activity (Figure S1C - half maximum value of 23.5 ms before saccade) required us to vary the width of the feedback signal with respect to the activity, so that feedback is initially broadly tuned, and prior to saccade onset, it becomes more focused. Such spatiotemporal pattern could well emerge by competitive interactions in occulomotor areas. Thus, $\sigma_{\mathrm{SA}}^{\mathrm{L}_1}$ in Equation 2 (main text) is now a function of time and for a 20° saccade (SA= 20) we use $\sigma_{\mathrm{SA}}^{\mathrm{L}_1} = \sigma_{20}^{\mathrm{L}_1}(t)$:

$$\sigma_{20}^{\mathrm{L}_1}(t) = \begin{cases} \frac{\sigma_{20}^{\mathrm{L}_1}(0)\gamma}{1+(\gamma-1)f(t)} & if \quad t \leq 0 \\ \sigma_{20}^{\mathrm{L}_1}(0) & else \end{cases}$$

where $\sigma_{20}^{\mathrm{L}_1}(0)$ is set to the value obtained by the fit of the spatial compression pattern (see Methods "Fitting procedure and parameters of the model" in main text) and $\gamma = \gamma_1$ (if $\epsilon(p_i^{\mathrm{L}_1}) \leq \epsilon(c^{\mathrm{ST}})$) and $\gamma = \gamma_2$ (if $\epsilon(p_i^{\mathrm{L}_1}) > \epsilon(c^{\mathrm{ST}})$) determines the width of the signal for $t = -\infty$. Note that $\epsilon(p_i^{\mathrm{L}_1})$ indicates the eccentricity of a given cell in a certain layer and $\epsilon(c^{\mathrm{ST}})$ indicates the eccentricity of the saccade target.

In particular, an initially broader population into the direction of larger eccentricities with respect to the saccade target ($\gamma_1 = 1$ and $\gamma_2 > 1$) avoids a strong and early mislocalization of the bar presented at 20°. At most, we were able to shift the half maximum value to 29.4 ms prior to saccade by broadening the shape of the early oculomotor signal ($\gamma_1 > 1$ and $\gamma_2 > 1$)(Figure S1D). An initially broader feedback signal leads to less compression in the near range of the saccade target early in time. However, by dropping the assumption that the gain increase is instantaneous, we can account also for earlier build-up activity (Figure S1E) where $\hat{r}_i^{\mathrm{L}_1}(t)$ (Equation 1, main text) is now $(\hat{r}_i^{\mathrm{L}_1}(t))^n$ with $n = 4$. At the first glance this might suggest we can tweak the model to fit any data, but the situation is different. Dropping the assumption of an instantaneous gain increase suggests that the source of the feedback signal can either originate from burst-like cells or from build-up cells. However, it also predicts that the effective signal depends on the burst activity, since the early build-up activity has only little impact on the gain. Taking the assumption of an instantaneous gain function, early feedback from visual cells activated by the presentation of the saccade target would not be consistent with the model (Figure S1F).

Feedback from open movement cells (simulated by $\sigma_{20}^{\mathrm{L}_1}(0) = \sigma_{20}^{\mathrm{L}_1}(0) \cdot k$ if $\epsilon(p_i^{\mathrm{L}_1}) > \epsilon(c^{\mathrm{ST}})$) impairs the data fit, since the population response from bars flashed at larger eccentricities do not get sufficiently distorted (Figure S1G).

It has also been observed that some cells still show significant activity after the end of the saccade, known as partially-clipped cells. A strong feedback signal during the saccade leads to stronger mislocalization effects into the direction of the saccade, since the center of the feedback signal moves with the eye relative to the stimulus location (Figure S1H). A summary of the parameters used in the above simulations is given in Table S1.

Table S1. Parameters of the simulations to predict the origin of oculomotor feedback in the brain.

Figure	$f(t)$	α	β	w	$\sigma_{20}^{\mathrm{L1}}$	$\sigma_{20}^{\mathrm{L2}}$	γ_1	γ_2	n	k
S2A	$f_g(t)$	25.0	12.0	15.0	0.4	3.5	2.0	5.0	1.0	1.0
S2B	$f_g(t)$	18.0	12.0	50.0	0.5	2.5	1.0	1.0	1.0	1.0
S1A	$f_e(t)$	0.095	0.13	30.0	0.51	4.7	1.0	1.0	1.0	1.0
S1B	$f_g(t)$	18.0	12.0	10.5	0.51	4.7	1.0	1.0	1.0	1.0
S1C	$f_g(t)$	20.0	12.0	10.5	0.51	4.7	1.0	3.0	1.0	1.0
S1D	$f_g(t)$	25.0	12.0	10.5	0.51	4.7	2.0	5.0	1.0	1.0
S1E	$f_g(t)$	39.0	24.0	10.5	1.0	9.5	2.0	5.0	4.0	1.0
S1F	$f_g(t)$	39.0	24.0	10.5	0.51	4.7	2.0	5.0	1.0	1.0
S1G	$f_g(t)$	18.0	12.0	10.5	0.51	4.7	1.0	1.0	1.0	4.0
S1H	$f_g(t)$	39.0	50.0	10.5	1.0	9.5	2.0	5.0	4.0	1.0
