# Supplementary material for: The Peri-Saccadic Perception of Objects and Space
Source: PLoS Comput Biol. 2008 Feb 15;4(2):e31. doi: 10.1371/journal.pcbi.0040031 (PMC2242822; doi:10.1371/journal.pcbi.0040031)
Supplement: Supplementary file 2 [file pcbi.0040031.sd002.pdf]

Supporting material for
Fred H. Hamker, Marc Zirnsak, Dirk Calow & Markus Lappe: The peri-saccadic perception of objects and space

Text S2: Estimates of the cortical areas involved based on receptive field size

Unfortunately, there are no sufficient quantitative receptive field measurements in human visual cortex, but it is likely that the receptive field size is larger than in monkeys [1]. Thus, we compared the required receptive field sizes of the model with the receptive field sizes from the areas of interest from monkey: V3a [2, 3], V4 [4–6], TEO [4], MT [7–10] (whereas most of the large receptive fields in MT have been reported by [10]), V2 [3, 11], LIP [12, 13], and TE [4]. The receptive fields of area TE are already quite large which might suggest that TE is only involved in object recognition. However, TE seems to contribute to spatial perception as well, since it is connected to LIPd and LIPv, similar as the projection from TEO [14].

References

1. Kastner, S., De Weerd, P., Pinsk, M. A., Elizondo, M. I., Desimone, R., Ungerleider, L. G. (2001) Modulation of sensory suppression: implications for receptive field sizes in the human visual cortex. *J. Neurophysiol.*, 86:1398-1411.

2. Galletti, C., Battaglini, P. P. (1989) Gaze-dependent visual neurons in area V3A of monkey prestriate cortex. *J. Neurosci.*, 9:1112-1125.

3. Nakamura, K., Colby, L. C. (2000) Visual, saccade-related, and cognitive activation of single neurons in monkey extrastriate area V3a. *J. Neurophysiol.*, 84:677-692.

4. Boussaoud, D., Desimone, R., Ungerleider, L. G. (1991) Visual topography of area TEO in the macaque. *J. Comp. Neurol.*, 306:554-575.

5. Desimone, R., Schein, J. S. (1987) Visual properties of neurons in area V4 of the macaque: sensitivity to stimulus form. *J. Neurophysiol.*, 57:835-868.

6. Gattass R., Sousa A. P. B., Gross C. G. (1988) Visuotopic organization and extent of V3 and V4 of the macaque. *J. Neurosci.*, 201:519-539.

7. Komatsu, H., Wurtz, R. H. (1988) Relation of cortical areas MT and MST to pursuit eye movements. I. Localization and visual properties of neurons. *J. Neurophysiol.*, 60:580-603.

8. Albright, T. D., Desimone, R. (1987) Local precision of visuotopic organization in the middle temporal area (MT) of the macaque. *Exp. Brain Res.*, 65:582-592.

9. Felleman, J. D., Kaas, J. H. (1984) Receptive-field properties of neurons in middle temporal visual area (MT) of owl monkeys. *J. Neurophysiol.*, 52:488-513.

10. Tanaka, K., Sugita, Y., Moriya, M., Saito, H. (1993) Analysis of object motion in the ventral part of the medial superior temporal area of the macaque visual cortex. *J. Neurophysiol.*, 69:128-142.

11. Gattass R., Gross C. G., Sandell J. H. (1981) Visual topography of V2 in the macaque. *J. Comp. Neurol.*, 201:519-539.

12. Blatt, G. J., Andersen, R. A., Stoner, G. R. (1990) Visual receptive field organization and cortico-cortical connections of the lateral intraparietal area (Area LIP) in the macaque. *J. Comp. Neurol.*, 299:421-445.

13. Ben Hamed, S., Duhamel, J. R., Bremmer, F. (2001) Representation of the visual field in the lateral intraparietal area of macaque monkeys: a quantitative recptive field analysis. *Exp. Brain Res.*, 140:127-144.

14. Webster, M. J., Bachevalier, J., Ungerleider, L. G. (1994) Connections of inferior temporal areas TEO and TE with parietal and frontal cortex in macaque monkeys. *Cereb. Cortex*, 4:470-483.
