# Supplementary material for: The Peri-Saccadic Perception of Objects and Space
Source: PLoS Comput Biol. 2008 Feb 15;4(2):e31. doi: 10.1371/journal.pcbi.0040031 (PMC2242822; doi:10.1371/journal.pcbi.0040031)
Supplement: Supplementary file 3 [file pcbi.0040031.sg001.pdf]

Supporting material for

Fred H. Hamker, Marc Zirnsak, Dirk Calow & Markus Lappe: The peri-saccadic perception of objects and space

Figure S1: Variation of the oculomotor feedback signal

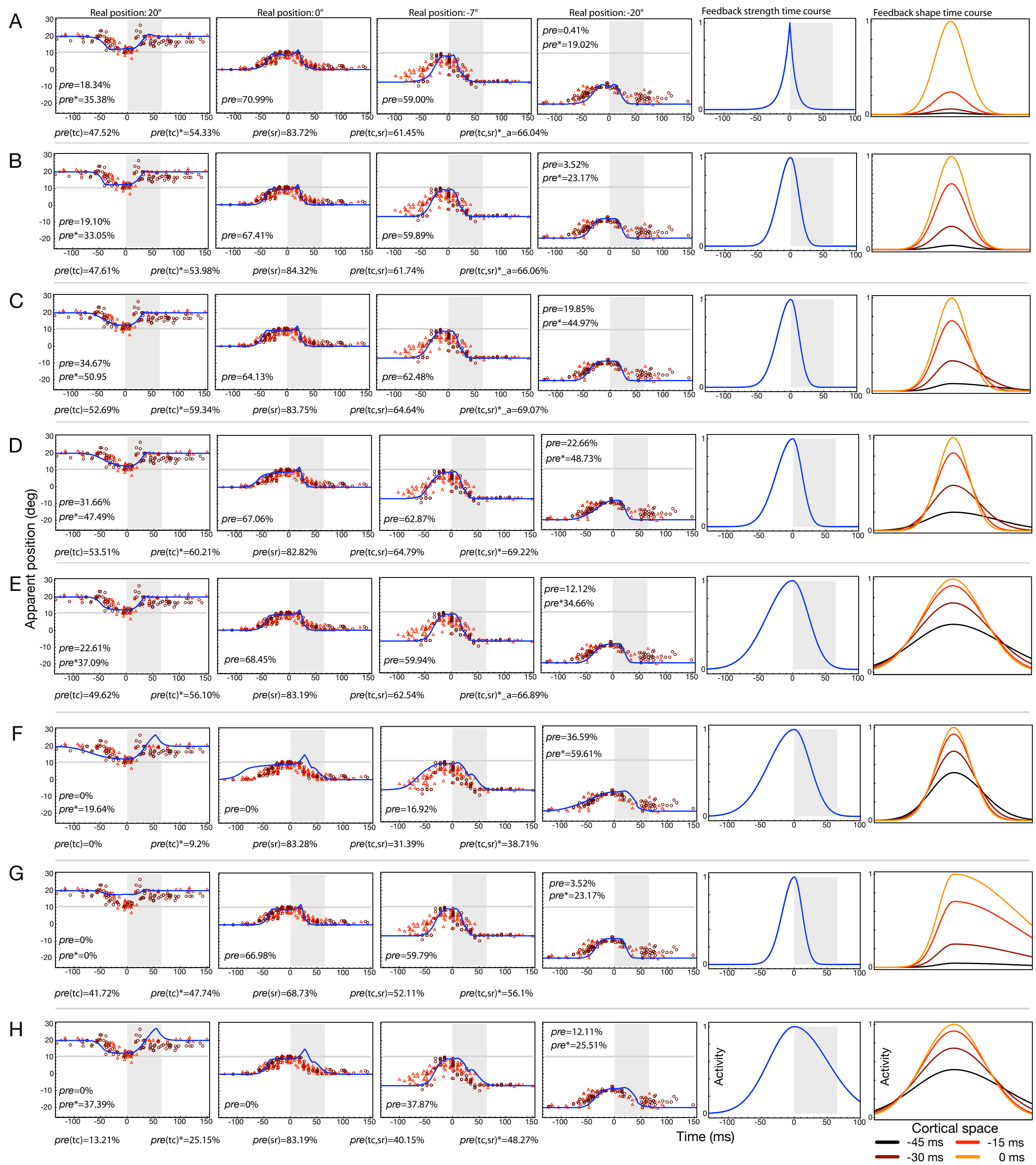

Figure S1. Details are given in Text S1. Variation of the oculomotor feedback signal in time and shape. In each case from left to right the time course of compression of four different bar positions, the time course of the feedback signal and different snapshots in time of the shape of the feedback signal are shown. For each data fit we determined the proportional reduction in error measure (*pre*). Since

the localization of the bar flashed at 20° and -20° shows a systematic mislocalization after saccade termination (shift in baseline) we also computed *pre** measures for these locations to determine the goodness of fit in the critical time range before and during a saccade. Below each panel we also give the aggregated *pre*-measures, where *pre*(sr) refers to the proportional reduction in error measure of the spatial range of compression, *pre*(tc) to the time course of compression, and *pre*(sc,tc) to a combined measure. A-E) Increase of the prelude activity in the feedback signal from late burst to build-up activity and the required changes in the shape of the feedback signal. F) Effect of a strong early visual component in the feedback signal using an instantaneous gain function. G) Effect of a strong contribution from cells with open movement fields. H) Effect of a strong contribution from unclipped cells in the feedback signal. The *pre*-values in Figure 3A have been taken from the simulation results shown here: Unclipped, H; Clipped, E; Open movement, G; Closed movement, C; Build-up (inst. gain), F; Build-up (damped gain), E; Burst (inst. gain), B.
