# Supplementary material for: The Peri-Saccadic Perception of Objects and Space
Source: PLoS Comput Biol. 2008 Feb 15;4(2):e31. doi: 10.1371/journal.pcbi.0040031 (PMC2242822; doi:10.1371/journal.pcbi.0040031)
Supplement: Supplementary file 4 [file pcbi.0040031.sg002.pdf]

Supporting material for

Fred H. Hamker, Marc Zirnsak, Dirk Calow & Markus Lappe: The peri-saccadic perception of objects and space

Figure S2: Variation of the receptive field sizes

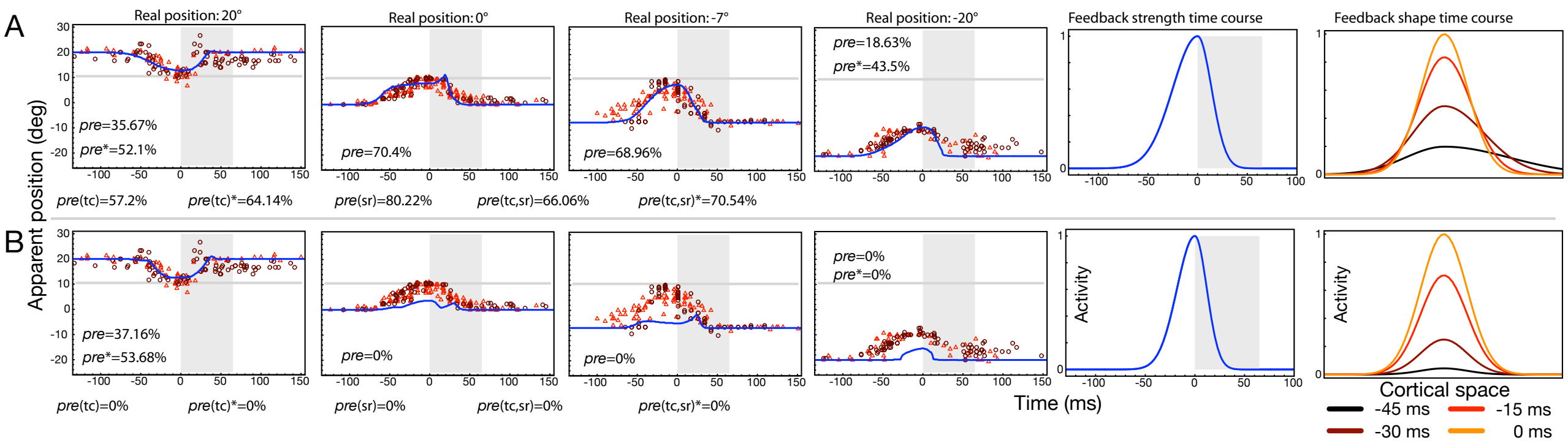

Figure S2. Variation of the receptive field sizes. For each data fit we determined the proportional reduction in error measure (*pre*). Below each panel we also give the aggregated *pre*-measures, where *pre*(sc) refers to the proportional reduction in error measure of the spatial range of compression, *pre*(tc) to the time course of compression, and *pre*(sc,tc) to a combined measure. In each case from left to right the time course of compression of four different bar positions, the time course of the feedback signal and different snapshots in time of the shape of the feedback signal are shown. A) Resulting data fit of the minimal receptive field size (solid line in Figure 3D). B) Resulting data fit using a too small receptive field size (dashed line in Figure 3D).
